# Humanizing pulmonary care in the era of acoustic artificial intelligence: toward global health equity

**DOI:** 10.3389/fmed.2025.1666820

**Published:** 2025-10-13

**Authors:** Bilal Irfan, Habeebah Muhammad-Kamal, Allison Newsome, Roberto Daniel Sirvent

**Affiliations:** ^1^Center for Bioethics, Harvard Medical School, Boston, MA, United States; ^2^Center for Surgery and Public Health, Brigham & Women's Hospital, Boston, MA, United States; ^3^Department of Neurology, University of Michigan Medical School, Ann Arbor, MI, United States; ^4^Department of Epidemiology, University of Michigan School of Public Health, Ann Arbor, MI, United States; ^5^Department of Global Health and Social Medicine, Harvard Medical School, Boston, MA, United States

**Keywords:** acoustic diagnostics, pulmonary care, artificial intelligence, health equity, algorithmic accountability, global health, acoustic AI, respiratory diagnostics

## Introduction

Artificial intelligence (AI) occupies an increasingly important position in pulmonary and neuromuscular medicine (1). Acoustic and voice-based AI systems are beginning to help clinicians detect respiratory and neuromuscular disease from coughs, breathing sounds, or short reading tasks, and they can at times enable remote monitoring that could outpace the capacity found in traditional clinics. Yet medicine's mandate extends beyond diagnosis and palliation, given that prevention, equity, and the defense of human dignity are also important responsibilities (2). This broader mandate invites a more exacting appraisal of contemporary AI practices, especially as they migrate from controlled laboratories to humanitarian crises, labor-exploitation zones, and communities already fractured by environmental violence. Drawing on recent developments in conflict medicine, disability studies, and algorithmic governance, we argue that the next decade of AI-assisted acoustic diagnostics must pivot from a narrow biomedical gaze to an agenda that prioritizes structural prevention, disability justice, and algorithmic accountability. Without such a pivot, the very systems designed to extend breath may instead replicate the patterns of suffocation—whether political, economic, or environmental—that have haunted respiratory health for generations. We aim to ground our arguments in the domain of bioethical, health, and legal considerations and the existing evidence and policy considerations.

## The clinical promise and its epistemic limits

The empirical advancements cataloged in recent literature support several recurrent claims. Machine learning can permit longitudinal surveillance outside tertiary centers, and expand the range of diagnostic accuracy within communities with limited clinical personnel. Improving access to high-quality healthcare not only advances person-centered care delivery, but also creates the foundation for which AI can bridge the persistent gap between clinical capacity and rising clinical demand.

Scholars examining work as a social determinant of health argue that ubiquitous mobile sensing and other “novel and creative methods of data collection” are now indispensable for tracking respiratory exposures and symptoms throughout workers' shifting schedules and tele-work days, thereby turning occupational epidemiology into a truly round-the-clock endeavor (3). Complementing that perspective, the American Thoracic and European Respiratory Societies describe how telemedicine infrastructures, from smartphone uploads to low-bandwidth video links, have begun to monitor migrants and refugees in conflict and border zones, enabling frontline teams to register day-to-day fluctuations in cough, medication access, and environmental exposures that would otherwise evade conventional clinic-based follow-up (4).

### Transparency

Transparent decision pathways are no longer an academic luxury but a practical and even juridical requirement. In July 2024, the New South Wales Dust Diseases Tribunal relied on pulmonologists' detailed explanations of spirometric and imaging evidence to award Craig Keogh AU$3.2 million for coal-dust-induced pneumoconiosis; the judgment hinged on experts' ability to trace restrictive ventilatory impairment to decades of particulate exposure in language comprehensible to legal fact-finders (5).

This precedent foreshadows what clinicians, and increasingly courts, will expect from algorithmic classifiers: when a model flags early fibrotic restriction, its salient acoustic or radiographic features must be presented in equally comprehensible terms, or the tool will struggle to earn the trust required for adoption.

### Accessibility and cost

Part of cultivating transparent systems involves ensuring accessibility and affordability, yet cost is often a crucial barrier to equitable AI implementation in healthcare. The Swaasa AI platform is a compelling example; with its ability to fulfill the unmet need for remote, cost-effective, limited specialist involvement in pulmonary tuberculosis treatment in geographically inaccessible communities (6).

### Bias

However, datasets can be an artifact of social history. Material instruments such as spirometers were standardized on the lung volumes of young, White men; when these norms were folded into workplace disability schemes, coal-miners whose readings fell short were branded as having inherent limitations rather than occupational injuries, allowing employers and insurers to withhold compensation (7).

Claims of model neutrality also evaporate on closer inspection. In thyroid ultrasound, for example, more than 80% of networks were trained on single-center Asian cohorts; when ThyNet, originally hailed for having an 89% accuracy, was re-tested on an external dataset its performance sank to 64%, illustrating how hidden sampling bias is embedded in the model and propagated through subsequent training pipelines (8).

Because supervised models optimize to reproduce training label patterns, they replicate and often conceal these embedded distortions. In fact, all 34 modeling studies identified in a disability-scoping review failed to audit their performance for subgroup bias or any other form of differential error (9). An epistemic boundary therefore emerges: high-fidelity pattern recognition cannot outrun low-fidelity ground truth. Until the discipline normalizes stratified performance reporting and participatory dataset curation, clinical brilliance will remain shadowed by statistical injustice.

## Humanitarian and conflict settings: AI respirators in a smoke-filled theater

Humanitarian emergencies, ranging from toxic industrial fires to the war-related bombardment of urban neighborhoods, generate a cascade of respiratory complaints that often out-strip on-site diagnostic capacity. In principle, low-cost acoustic classifiers trained to recognize cough timbre, stridor, or early laryngeal edema could provide clinicians with a “first-pass” triage signal when radiography or bronchoscopy are unavailable. In these settings, high particulate loads, sirens, crowd noise, and heterogeneous handsets may also serve to intensify channel mismatch, making noise-robust training and prospectively reported noise-stratified metrics prerequisites rather than niceties.

Yet, recent experience with algorithmic decision-making in insurance markets underscores how easily pattern-recognition can be redirected from patient care to cost control. Class-action pleadings against UnitedHealth, Humana, and Cigna describe proprietary models that reviewed and rejected hundreds of thousands of claims in bursts lasting only seconds; one filing alleges that Cigna's clinicians “signed off” on more than 300,000 denials in a 10-week window, an interval that averages 1.2 s per case and is feasible only through automated scoring (10).

There are also notable patterns of structural bias. For example, in a cohort of 1.5 million privately-insured patients seeking services that should be free under the Affordable Care Act, preventive-care claims were 43% more likely to be refused for households earning under $30,000 and patients classified racially as Asian, Hispanic, or Black faced denial rates two- to 3-fold higher than their counterparts classified racially as White in the United States (11).

These patterns have direct justice-orientated clinical implications. Humanitarian agencies increasingly purchase AI-as-a-service from the same vendors that dominate domestic insurance analytics. Without enforceable safeguards, a cough-based triage model deployed in a refugee clinic could inherit the same optimization logic used in the insurance industry: identify high-cost cases quickly and channel scarce medication elsewhere, regardless of clinical necessity. Historical precedent warns of what follows when technology quantifies harm but power suppresses redress. After U.S. nuclear tests in the Marshall Islands, radioactive iodine damaged the thyroids of local musicians; decades later many remain voiceless and uncompensated despite well-documented causal links (12). Recording dysphonia with state-of-the-art AI would not, by itself, address that inequity.

Acoustic AI is also bounded by practical and deployment constraints that can attenuate performance outside controlled settings. Auscultation quality remains sensitive to device characteristics and user expertise, and even among digital stethoscopes detection performance varies by device, suggesting that robust applications of it can depend on substantial, high-quality data and rigorous validation (1). More broadly, lack of standardized data and interoperability, data-protection and privacy concerns, and variable institutional readiness can limit real-world uptake, reinforcing the need for widely accepted standards in clear language and ongoing human oversight in clinical use. To avoid over-idealizing capability, acoustic systems could be evaluated under realistic conditions and report device-specific performance with deployment plans that include quality checks and clinician review.

A route forward is outlined in human-computer-interaction scholarship that brought together technologists, field workers, and policy leads to co-design an “ECHO” governance architecture, Educate, Co-create, Hand-hold, Optimize, for any AI introduced into relief operations (13). The framework couples participatory design with mandatory audit trails and shared data stewardship to ensure that affected communities, not only distant donors or contractors, retain oversight of model purpose and impact. Binding this architecture to legal rights of explanation, appeal, and material remedy is essential if acoustic AI, and the many other algorithms now migrating into crisis medicine, are to become instruments of solidarity rather than tools for rationing.

Nevertheless, some signs of potential benefits have also emerged from various settings around the world. In Malawi, an AI-enabled digital auscultation system for children (2–59 months) hospitalized with WHO-defined severe pneumonia achieved 83.1% agreement at the chest-position level and 91.6% at the patient level with a trained physician listening panel in a high-noise ward setting, thus supporting the feasibility of use even in noisy environments when coupled with human oversight of recording quality (14).

## Labor, environment, and the preventive horizon

Respiratory disease is deeply entangled with social production. Informal waste workers inhale toxic bioaerosols, nail salon technicians absorb volatile compounds, and agricultural migrants labor amid pesticide drift. Occupational health literature has documented these exposure patterns' links to restrictive and obstructive syndromes, yet surveillance remains inadequate (15).

Occupation-related respiratory disease is not confined to heavy industry; a recent systematic review shows that sanitation workers bear a disproportionate disease burden (15). Across 4,521 sanitary workers in 11 countries, a review found a pooled prevalence of occupation-related respiratory disease of 32.6%, with street sweepers the most affected subgroup at 36.4% (15).

The problem is sharply stratified by national income: prevalence averaged 35.2% in low-income settings vs. 20% in high-income ones, a gap that was attributed to routine contact with bioaerosols, dust, and toxic residues in the absence of adequate protective equipment or safety oversight (15). Reported clinical outcomes ranged from cough and wheeze to chronic obstructive pulmonary disease, and were consistently linked to modifiable workplace factors such as a lack of masks, long shifts, and minimal occupational-health training (15).

Machine learning can potentially improve occupational health surveillance. Edge devices that record brief cough episodes during a factory shift may help feed federated models detecting early pneumoconiosis. Satellite aerosol data fused with community-submitted voice logs might assist in mapping exposure hotspots, offering regulators near real-time evidence. If such systems had existed in the Mississippi River petrochemical corridor, where asthma and chronic lung disease rates tower, residents might have demonstrated causality sooner, forestalling new permits and compelling remediation.

Prevention, however, demands more than detection. Medicine's mandate extends to altering the conditions that give rise to disease. If AI surfaces exposure, yet employers respond solely with disposable masks, the political economy of sacrifice remains untouched. A preventive agenda must therefore couple model outputs to enforceable workplace standards, statutory compensation, and, where necessary, industrial phase-out. It must also confront militarized origins of toxics. Agent Orange continues to scar respiratory and endocrine health among Vietnamese civilians and those involved in military activity there (16–18); current Pentagon partnerships with major technology firms raise concern that new chemical or biological agents will be algorithmically engineered. An AI community committed to pulmonary health could advocate for international treaties limiting data-driven weapons design and for reparative healthcare funding in previously targeted regions.

## Discussion

Acoustic AI possesses extraordinary capacity to detect respiratory and neuromuscular disease with speed and precision unknown in previous eras. Yet these same systems, if left unexamined, risk reproducing the harms of racial capitalism, militarism, ableism, and algorithmic austerity. Preventive medicine offers a pathway through the paradox. By orienting AI toward the conditions that injure lungs and vocal cords initially, clinicians and technologists can align innovation with medicine's foundational goals: to prevent disease, to relieve suffering, and to promote justice.

Achieving this alignment requires a comprehensive multi-tiered clinical program. Data provenance must be decolonized, centering historically excluded voices and marking the sociopolitical context of each recording. Bias auditing must become as routine as calibration curves, with performance reported across intersectional categories. Regulatory bodies should treat biased respiratory algorithms as patient safety threats, subject to recall.

In practical terms, decolonizing datasets requires shifting governance, labor, and benefits to the communities whose voices populate the corpus. Steps can be broken down by different groups and actors operating in the space into short term, medium term, and long term goals and pivots as meets their needs or specific tools. First, establishing community-participatory annotation protocols might be worth considering: recruit and compensate local annotators; constitute a community review board with input over labels and metadata; and co-author dataset documentation with those stakeholders. Second, it is possible to consider adopting community data agreements that recognize data sovereignty (including “no export” and “no secondary use” clauses unless re-consented), mandate results-return to clinics, and earmark revenue or grant overhead for local health services. Third, it may be worth considering the implementation of cross-regional, collaboratively governed federated learning so models train where data reside: possibly using secure aggregation and periodic cross-site evaluation; setting site-level fairness constraints and publishing stratified performance (sex, age, language/dialect, disability, device class); and rotating stewardship across partner institutions to avoid a single-center epistemic monopoly. Fourth, releasing datasheets/model cards that record sociopolitical context of collection, annotator demographics, pay, and known failure modes, and pre-registering audit plans so communities can trigger remedial actions when harms are detected may be avenues to explore. Together, these measures might be able to aid in converting “decolonization” from rhetoric to a somewhat verifiable workflow, aligning aspects of the technical practice with equity commitments (19).

Humanitarian deployments must be governed by transparent protocols that guarantee equitable treatment distribution, prohibit surveillance repurposing, and ensure local ownership of data. Insurance algorithms must be auditable in real time, with patient-friendly appeals and strict penalties for unjust denials.

Finally, education in pulmonary medicine and computer science must integrate disability justice, environmental health, and the history of medical racism so that future professionals recognize the sociogenic roots of the breath.

If those measures are adopted, acoustic AI could help dismantle sacrifice zones, alert regulators to industrial poisoning, and ensure rapid care even in bombed hospitals. It could amplify voices of tracheostomised poets, document labor abuse, and secure reparations for communities scarred by nuclear fallout. In short, it could extend the radius of breathable justice. The alternative is already visible in the algorithms that silence claims, misclassify dark-skinned hypoxia, and encode old hierarchies in new code (20).

Medicine now stands at a threshold. The task is not to celebrate or to condemn AI, but to harness it within a larger movement for planetary health and human dignity. The right to breathe, after all, is a precondition for every other right we hold dear.

## References

[1] Karthika M Sreedharan JK Shevade M Mathew CS Ray S. Artificial intelligence in respiratory care. Front Digit Health. (2024) 6:1502434. 10.3389/fdgth.2024.1502434 39764208 PMC11700984

[2] Irfan B Jaber B Awwad M AlSourani T Shammala AA Irfan B. Examining Contemporary human dignity: how human rights evolve and reshape health justice. Cureus. (2025) 17:82316. 10.7759/cureus.82316 40376355 PMC12080955

[3] Frank J Mustard C Smith P Siddiqi A Cheng Y Burdorf A. Work as a social determinant of health in high-income countries: past, present, and future. Lancet. (2023) 402:1357–67. 10.1016/S0140-6736(23)00871-1 37838441

[4] Roman J Viegi G Schenker M Ojeda VD Pérez-Stable EJ Nemery B. Research needs on respiratory health in migrant and refugee populations. An official American Thoracic Society and European Respiratory Society workshop report. Ann ATS. (2018) 15:1247–55. 10.1513/AnnalsATS.201807-478ST 30382778 PMC12392160

[5] Press AA. Ex-coalminer Awarded $3.2m for Black Lung in Australian First. The Guardian (2024). Available online at: https://www.theguardian.com/australia-news/article/2024/jul/25/coal-miner-craig-keogh-black-lung-case-payout (Accessed July 11, 2025).

[6] Yellapu GD Rudraraju G Sripada NR Mamidgi B Jalukuru C Firmal P. Development and clinical validation of Swaasa AI platform for screening and prioritization of pulmonary TB. Sci Rep. (2023) 13:4740. 10.1038/s41598-023-31772-9 36959347 PMC10034902

[7] Liao S Carbonell V. Materialized oppression in medical tools and technologies. The Am J Bioethics. (2023) 23:9–23. 10.1080/15265161.2022.2044543 35262465

[8] Lu Q Wu Y Chang J Zhang L Lv Q Sun H. Application progress of artificial intelligence in managing thyroid disease. Front Endocrinol (Lausanne). (2025) 16:1578455. 10.3389/fendo.2025.1578455 40600013 PMC12208828

[9] El Morr C Kundi B Mobeen F Taleghani S El-Lahib Y Gorman R. AI and disability: a systematic scoping review. Health Informatics J. (2024) 30:14604582241285743. 10.1177/14604582241285743 39287175

[10] Schreiber M. New AI Tool Counters Health Insurance Denials Decided by Automated Algorithms. The Guardian (2025). Available online at: https://www.theguardian.com/us-news/2025/jan/25/health-insurers-ai (Accessed July 11, 2025).

[11] Hoagland A Yu O Horný M. Social determinants of health and insurance claim denials for preventive care. JAMA Netw Open. (2024) 7:e2433316. 10.1001/jamanetworkopen.2024.33316 39292461 PMC11411384

[12] In Marshall Islands, Radiation Radiation Threatens Tradition of Handing Down Stories by Song. Los Angeles Times (2019). Available online at: https://www.latimes.com/projects/marshall-islands-radiation-effects-cancer/ (Accessed July 11, 2025).

[13] Bhatnagar T Omar M Orlic D Smith J Holloway C Kett M. Bridging AI and Humanitarianism: an HCI-Informed Framework for Responsible AI Adoption. In: Proceedings of the 2025 CHI Conference on Human Factors in Computing Systems. CHI '25. New York, NY: Association for Computing Machinery (2025). p. 1–17. 10.1145/3706598.3713184

[14] Hoekstra NE Chagomerana MB Smith ZH Kala A McLane I Verwey C. Performance of an artificial intelligence algorithm for interpreting lung sounds from children hospitalised with pneumonia in Malawi. J Glob Health. 15:04264. 10.7189/jogh.15.04264 40968644 PMC12447016

[15] Tolera ST Alemu B Mengistu DA Deressa A. Occupation-related respiratory diseases among sanitary workers in the workplace: a systematic review and meta-analysis. Front Public Health. (2024) 12:1501768. 10.3389/fpubh.2024.1501768 39659714 PMC11628511

[16] Bernicker EH. Agent orange and cancer. In: Environmental Oncology. Cham: Springer (2023). p. 289–303. 10.1007/978-3-031-33750-5_12

[17] Kaul B Lee JS Glidden DV Blanc PD Zhang N Collard HR. Whooley MA. Agent orange exposure and risk of idiopathic pulmonary fibrosis among US veterans. Am J Respir Crit Care Med. (2022) 206:750–7. 10.1164/rccm.202112-2724OC 35559726 PMC9799114

[18] Cohn M. Vietnamese Agent Orange Victims Have Never Been Compensated. Rashida Tlaib Wants to Change That. Truthout (2025). Available online at: https://truthout.org/articles/vietnamese-agent-orange-victims-remain-uncompensated-tlaib-aims-to-change-that/ (Accessed July 11, 2025).

[19] Mohamed S Png M-T Isaac W. Decolonial AI: decolonial theory as sociotechnical foresight in artificial intelligence. Philos Technol. (2020) 33:659–84. 10.1007/s13347-020-00405-8

[20] Sjoding MW Ansari S Valley TS. Origins of racial and ethnic bias in pulmonary technologies. Annu Rev Med. (2023) 74:401–12. 10.1146/annurev-med-043021-024004 35901314 PMC9883596

